# CARDIOVASCULAR RISK FACTORS AND MENTAL HEALTH OUTCOMES AMONG LGB+ OLDER ADULTS

**DOI:** 10.1093/geroni/igae098.3818

**Published:** 2024-12-31

**Authors:** Jennifer Smith, Robert Rodriguez, Joel Anderson

**Affiliations:** University of Tennessee, Knoxville, Knoxville, Tennessee, United States; University of Texas Medical Branch: School of Public and Population Health, Galveston, Texas, United States; University of Tennessee-Knoxville, Knoxville, Tennessee, United States

## Abstract

Emerging evidence highlights that sexual and gender minority (SGM) adults exhibit a higher prevalence of cardiovascular disease (CVD) and its risk factors compared with their non-SGM peers. This increased susceptibility underscores a critical intersection with cognitive health given the link between CVD and cognitive decline and early-onset dementia. Additionally, mental health challenges such as depression and anxiety not only heighten CVD risk but also predict severe cardiovascular complications. Utilizing cross-sectional data from the National Health Interview Survey (NHIS), this study examined these interconnected health issues among older adult LGB+ populations to inform comprehensive interventions aimed at addressing both cardiovascular and cognitive health. This cross-sectional study analyzed data from the 2022 NHIS data to describe cardiovascular and cognitive health characteristics among LGB+ and non-LGB+ older adults using chi-squared tests. The sample was limited to adults who self-reported being age 50 years or older. A CVD risk variable was created based on Framingham criteria. Data from 13,426 participants, including 341 LGB+ individuals, revealed significant differences in demographic characteristics, health, and lifestyle. LGB+ older adults were younger and more frequently formally educated. They exhibited lower rates of CVD risk factors and increased physical activity and preventative health behaviors, yet higher incidence of depression and anxiety and higher rates of smoking and alcohol use. These preliminary findings highlight the unique characteristics of LGB+ older adults in terms of cardiovascular health, offering potential targets for culturally tailored interventions.

